# A Comparison of Shallow and Deep Learning Methods for Predicting Cognitive Performance of Stroke Patients From MRI Lesion Images

**DOI:** 10.3389/fninf.2019.00053

**Published:** 2019-07-31

**Authors:** Sucheta Chauhan, Lovekesh Vig, Michele De Filippo De Grazia, Maurizio Corbetta, Shandar Ahmad, Marco Zorzi

**Affiliations:** ^1^School of Computational and Integrative Sciences, Jawaharlal Nehru University, New Delhi, India; ^2^TCS Research, New Delhi, India; ^3^Department of General Psychology, Padova Neuroscience Center, University of Padova, Padua, Italy; ^4^Department of Neurosciences, Padova Neuroscience Center, University of Padova, Padua, Italy; ^5^Department of Neurology, Washington University School of Medicine, St. Louis, MO, United States; ^6^IRCCS San Camillo Hospital, Venice, Italy

**Keywords:** deep learning, machine learning, stroke, cognitive deficit, magnetic resonance imaging, brain lesion

## Abstract

Stroke causes behavioral deficits in multiple cognitive domains and there is a growing interest in predicting patient performance from neuroimaging data using machine learning techniques. Here, we investigated a deep learning approach based on convolutional neural networks (CNNs) for predicting the severity of language disorder from 3D lesion images from magnetic resonance imaging (MRI) in a heterogeneous sample of stroke patients. CNN performance was compared to that of conventional (shallow) machine learning methods, including ridge regression (RR) on the images’ principal components and support vector regression. We also devised a hybrid method based on re-using CNN’s high-level features as additional input to the RR model. Predictive accuracy of the four different methods was further investigated in relation to the size of the training set and the level of redundancy across lesion images in the dataset, which was evaluated in terms of location and topological properties of the lesions. The Hybrid model achieved the best performance in most cases, thereby suggesting that the high-level features extracted by CNNs are complementary to principal component analysis features and improve the model’s predictive accuracy. Moreover, our analyses indicate that both the size of training data and image redundancy are critical factors in determining the accuracy of a computational model in predicting behavioral outcome from the structural brain imaging data of stroke patients.

## Introduction

Deep learning methods have gained popularity because they often outperform conventional (i.e., shallow) machine learning methods and can extract features automatically from raw data with little or no preprocessing (LeCun et al., 2015). Among the many implementations of deep learning models, convolutional neural networks (CNNs) (Krizhevsky et al., 2012) are particularly suited for medical imaging data (Shen et al., 2017). A prominent example is the recent demonstration that a CNN trained end-to-end from pixels of medical images to disease labels in a skin cancer classification problem performed at the level of expert dermatologists (Esteva et al., 2017). Deep learning has also been applied to neuroimaging data for brain-based classification of psychiatric and neurological disorders (Arbabshirani et al., 2017; Vieira et al., 2017). For example, several studies have tackled the diagnosis of Alzheimer’s disease and its prodromal stage (mild cognitive impairment) using magnetic resonance imaging (MRI) data as input to a CNN (for comprehensive overviews see Arbabshirani et al., 2017; Vieira et al., 2017).

**FIGURE 1:**
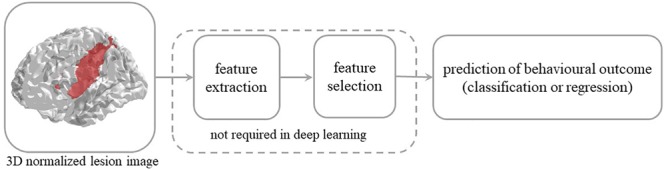
Steps involved in the prediction of behavioral outcome in stroke patients from 3D lesion images. Conventional machine learning methods typically rely on data preprocessing and feature selection, which are dispensed with in deep convolutional neural networks.

The use of deep learning on neuroimaging data is particularly interesting because MRI scans produce 3D images. Though some studies have used 2D slices of the brain volume as independent images for training, state-of-the-art deep learning techniques and massive use of GPU-computing allow to feed a whole 3D image to a CNN, despite the very high dimensional input, without decomposition or preprocessing. In the present work we employ a 3D CNN framework in the context of predicting behavioral outcomes of stroke patients from MRI lesion images. The latter can be formalized as a regression problem, where the learning objective is to map the 3D image of a patient’s brain lesion to the real-valued score representing the behavioral performance of the same patient (see Figure 1). This problem has been previously tackled with conventional machine learning methods (Price et al., 2010; Hope et al., 2013; Zhang et al., 2014; Corbetta et al., 2015; Siegel et al., 2016) but not with deep learning. Moreover, the use of deep learning for regression (rather than classification) problems in clinical neuroimaging is still sparse (Vieira et al., 2017).

A widely shared assumption in cognitive neurology and neuropsychology is that the effect of brain damage on behavior and cognition depends on location and size of the lesion. This has led to the long-standing and systematic effort to identify the relationship between brain structure and function. Specifically, the vast majority of studies have sought to establish which brain lesion is associated to a specific (categorically defined) deficit (Rorden and Karnath, 2004). The latter mapping is reversed when the attempt is to predict behavioral performance from lesion information and the task is more challenging because it implies that lesion-behavior relationships are consistent across individuals and can be used to predict behavior in new patients (Price et al., 2017). However, the consistency of the association is questionable because it depends on multiple factors and non-linear interactions might be present in the data, thereby calling for a machine learning approach to this problem (Chen et al., 2008; Hope et al., 2013; Smith et al., 2013; Zhang et al., 2014; Siegel et al., 2016; Price et al., 2017). Notably, conventional machine learning methods typically require extraction and selection of image features that represent topological information about the lesion, a critical step that is dispensed with in the deep learning approach.

In the present study, we take advantage of the data from a relatively large and heterogeneous cohort of stroke patients (Corbetta et al., 2015) to investigate the feasibility of a deep learning model for predicting behavioral performance from lesion images. We focused on the prediction of language scores, in line with several previous studies that examined lesion-behavior relationship in stroke patients using machine learning (Price et al., 2010; Hope et al., 2013, 2015; Zhang et al., 2014). Language deficits are a very frequent outcome of stroke (particularly following left hemisphere damage) and their neural correlates show lower inter-individual variability in comparison to other cognitive functions like memory (Siegel et al., 2016). Moreover, the prospect of predicting the functional recovery of language has profound implications for clinical practice (Price et al., 2010).

**FIGURE 2:**
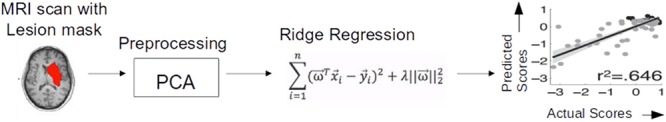
Ridge regression method used by Siegel et al. (2016) for predicting behavioral deficits in stroke patients from MRI lesion images.

The main aim of the present study was therefore to assess the CNN/deep learning approach against conventional (i.e., shallow) machine learning methods. Shallow machine learning has been previously applied on the current stroke dataset using multivariate ridge regression (RR) trained on features of the lesion images extracted by principal component analysis (PCA) to predict patients’ behavioral outcomes (Corbetta et al., 2015; Siegel et al., 2016). The resulting model (hereafter PCA + RR, see Figure 2 and “Materials and Methods” section for details), when trained and evaluated on the language deficit scores using leave-one-(patient)-out cross-validation, accounted for about 60% of the variance (using *r*^2^ as goodness-of-fit measure). This method, re-implemented in the present study, provides a useful benchmark for comparative evaluation of the deep learning approach. Moreover, we broadened the comparison between deep and shallow machine learning techniques by testing a kernel-based approach (Vapnik, 1998), that is support vector regression (SVR). A SVR-based approach has been previously proposed in the context of multivariate lesion-symptom mapping in stroke patients (Zhang et al., 2014), where the SVR model’s ability to predict language deficit scores on the patients’ sample from the lesion image features was also tested (though with relatively poor fit).

A complementary aim of the study was to examine how the different machine learning approaches are affected by the number of patient cases available for training and by the diversity of lesions in the sample. State-of-the-art stroke studies typically include a small number of patients (order of 100; see Corbetta et al., 2015) in comparison to publicly available databases of patients suffering from other neurological conditions such as Alzheimer’s disease (but see Price et al., 2010) This raises the question of whether the amount of data is adequate for a deep learning approach and, more generally, how performance of the different machine learning methods scales with the size of the training database. Indeed, limited sample size has been identified as the main bottleneck for neuroimaging-based prediction of brain disorders (see Arbabshirani et al., 2017, for review and discussion). Second, we investigated the role of redundancy in the image database, that is the similarity between a given test image and the images used to train the model. We therefore assessed to what extent these two factors are critical in determining the predictive accuracy of the different machine learning models.

## Materials and Methods

### Dataset

The dataset was obtained from a study on stroke patients carried out at the Washington University School of Medicine. The study and all procedures were approved by the Washington University School of Medicine Internal Review Board; written informed consent was obtained from all participants in accordance with the Declaration of Helsinki. Subject enrolment, inclusion and exclusion criteria, and demographic information are described in detail in Corbetta et al. (2015); in brief, the study included 132 stroke patients (mean age 52.8 years with range 22–77; 119 right handed, 63 female, 64 right hemisphere damage), recruited through the inpatient service at Barnes-Jewish Hospital and the Rehabilitation Institute of St. Louis. Imaging and behavioral testing session were usually performed on the same day. Patient scanning was performed on a Siemens 3T Tim-Trio. Structural scans consisted of a sagittal MP-RAGE T1-weighted image (TR = 1950 ms, TE = 2.26 ms, flip angle = 9°, voxel size = 1.0 × 1.0 × 1.0 mm, slice thickness = 1.00 mm), a transverse turbo spin-echo T2-weighted image (TR = 2500 ms, TE = 435 ms, voxel-size = 1.0 × 1.0 × 1.0 mm, slice thickness = 1.00 mm), and a sagittal FLAIR (fluid attenuated inversion recovery) (TR = 7500 ms, TE = 326 ms, voxel-size = 1.5 × 1.5 × 1.5 mm, slice thickness = 1.50 mm). Individual T1 MRI images were registered to the Montreal Neurological Institute (MNI) brain using FSL (FMRIB Software Library) FNIRT (FMRIB non-linear imaging registration tool) (Andersson et al., 2007). Lesions were manually segmented on individual structural MRI images (T1-weighted MPRAGE, T2-weighted spin echo images, and FLAIR images) using the Analyze biomedical imaging software system (Robb and Hanson, 1991). Two board-certified neurologists reviewed all segmentations.

Though the original dataset includes behavioral data for multiple cognitive domains (e.g., language, memory, attention), in the present study we focused on predicting performance in the language domain. As noted in the section “Introduction,” the rationale for this choice was threefold: (i) a language impairment is the most common cognitive deficit following stroke (typically when causing left hemisphere damage); (ii) language is the cognitive domain in which a shallow machine learning method has achieved the highest predictive accuracy on the same dataset (Siegel et al., 2016); and (iii) the problem of predicting language deficit scores from lesion images has been attempted with different methods and by different research groups (Hope et al., 2013; Zhang et al., 2014; Siegel et al., 2016).

Our dataset included all patients who had MRI lesion images and language scores available (*N* = 98), which is the same sample previously used by Siegel et al. (2016) to develop their RR method (see below for further details). The data for each patient consisted of a 3D image of the lesion with a 3 mm isovoxel resolution normalized the MNI coordinate space (61 × 73 × 61 voxels). The current image resolution limits the computational burden implied by the large-size 3D image space and it is fully adequate for representing the spatial topography of the lesions. The language score of each patient summarized performance across several language tasks as it captured their shared variance (first principal component) and it was normalized to represent impaired performance with negative values (Siegel et al., 2016). Accordingly, 29 of the stroke patients presented with a language deficit.

### Ridge Regression Method

We re-implemented the RR method and used its performance as a baseline for assessing the other methods. The pipeline used in the original study (Siegel et al., 2016) is illustrated in Figure 2. Lesion images were first preprocessed using PCA to strongly reduce the high dimensionality of the image space. Here, we replicated the PCA preprocessing step using singular value decomposition (SVD) in python using scikit-learn module. The first 56 components explained 95% of variance and were retained as input features for RR. The latter is a method for modeling the relationship between a scalar dependent variable *y* (output) and one or more explanatory variables denoted by *x* (input). RR differs from multiple linear regression because it uses L2-normalization for regularization of model coefficients, so that unimportant features are automatically down weighted or eliminated, as given in the cost function below:

$$\begin{array}{c} \overset{n}{\underset{i=1}{\Sigma }}{\left(w^{T}x_{i}-y_{i}\right)}^{2}+\lambda ||w|{|}_{2}^{2} \end{array}$$

where *n* is the number of subjects, *w* is the weight vector that describes the relative importance of each feature in *x* to the prediction of *y*, and λ is the regularization coefficient. Optimal weights were computed across the entire training set using gradient descent to minimize error for the RR equation. Training and testing was carried out using a leave-one-out cross validation (LOOCV) loop (Golland and Fischl, 2003), in which one patient is left out from training at a time (cycling through all patients) and used only for testing. In each loop, the regularization coefficient lambda was optimized by identifying a lambda between λ = 1 and 150 that minimized leave-one-out (LOO) prediction error over the entire training dataset. The optimized lambda was λ = 100 for all LOOCV cycles. Predictions on the left-out test data were pooled and the model accuracy was assessed using the square of the Pearson correlation coefficient between actual and predicted behavioral scores (Siegel et al., 2016). RR in this work was implemented in python (scikit-learn module), using linear least square function as loss function and L2-normalization for regularization.

### Support Vector Regression

To broaden the comparison between deep and shallow machine learning techniques, we also implemented a kernel-based approach to predicting behavioral scores from brain lesion images. SVR is a kernel-based learning machine for regression (Vapnik et al., 1997). Instead of minimizing the observed training error, SVR attempts to minimize the generalization error bound. SVR can be thought of as a linear regression function in a high dimensional feature space where the input data are mapped via a non-linear function (Cortes and Vapnik, 1995; Smola and Schölkopf, 2004).

Considering a training dataset {(x_1_, y_1_),(x_2_, y_2_),… (x_l_, y_l_)}⊂ R^n^ × R, the following function is estimated in SVR for linear regression:

$$\begin{array}{c} f(x)=\langle w,x\rangle +b;\mathrm{where}:w,x\in R^{n},b\in R \end{array}$$

by minimizing the so-called regularized risk functional (Vapnik et al., 1997; Vapnik, 1998; Basak et al., 2007) $\frac{1}{2}$||w||^2^ + C ⋅ R_emp_[f].

The first term $\frac{1}{2}$||w||^2^ is called the regularization term. Minimizing this term will make the function as flat as possible. The second term R_emp_[f] is the empirical error measured by the loss function and *C* is called the regularization constant which determines tolerated deviations from the loss function. In this problem, we used 𝜖 – insensitive loss function L_𝜖_:

$$\begin{array}{c} L_{\epsilon }(y_{i},f(x_{i}))=max\{0,|y-f(x)|-\epsilon \} \end{array}$$

This defines 𝜖 tube, so that if the predicted value is within the tube the loss is zero, while if the predicted point is outside the tube, the loss is the magnitude of the difference between the predicted value and the radius 𝜖 of the tube. Slack variables ξ,ξ^∗^ are used to deal with infeasible constraints of the optimization problem. Then the problem can be formulated as:

$$\begin{array}{c} \min\frac{1}{2}||w|{|}^{2}+C\overset{l}{\underset{i=1}{\Sigma }}(\xi_{i},\xi_{i}^{*}) \end{array}$$

subject to

$$\begin{array}{c} subject to\left\{ \begin{array}{c} y_{i}-\langle w,x_{i}\rangle -b\leq \epsilon +\xi_{i}\\ \langle w,x_{i}\rangle +b-y_{i}\leq \epsilon +\xi_{i}^{*}\\ \xi_{i},\xi_{i}^{*}\geq 0 \end{array}\right. \end{array}$$

The purpose is to construct a Lagrange function from the objective function and the corresponding constraints, by introducing a dual set of variables (Lin et al., 2006). The constant C > 0 determines the trade-off between the flatness of f and the amount up to which deviations larger than 𝜖 are tolerated. In cases where non-linear functions are optimized, it is performed by mapping the input space x_i_ into higher dimensional space through function ϕ(x_i_), which linearises the relationships between x_i_ and y_i_. A kernel function *K* is used to simplify the mapping. By using the kernel function, the data can be mapped implicitly into a feature space (without full knowledge of ϕ), which is therefore very efficient (Schölkopf and Smola, 2002; Lin et al., 2006). In this work we only used a radial basis function (RBF) kernel, which is defined as follows:

$$\begin{array}{c} K(x_{i},x)=exp(-\gamma ||x-x_{i}|{|}^{2}) \end{array}$$

The SVR simulations were based on the libSVM framework implemented in python using sci-kit learn module. We trained our model and tested its performance using LOO cross-validation on the full dataset. The learning parameters were set to *C* = 50 and 𝜖 = 0.1 (note that large value of 𝜖 generally gives large errors in the solution), whereas the RBF kernel coefficient γ was set to the reciprocal of the number of input features (i.e., the default value in the SVR implementation).

### Convolutional Neural Networks

Convolutional neural networks exploit spatially local correlation by enforcing a local connectivity pattern between neurons of adjacent layers. CNN performs image classification by discovering low level features (such as edges and curves) and then building up to more abstract representations through a series of convolutional layers. A typical CNN architecture consists of at least four different layers namely convolutional layer, pooling/subsampling layer, fully connected layer, and an output layer, as explained below.

#### Convolutional Layer

It comprises of a set of filters, each independently convolved with the image. These filters (or kernels) have a small receptive field but extend through the full depth of the input volume. During the forward pass, each filter is convolved across the width and height of the input volume, computing the dot product between the entries of the filter and the input and producing a 2D activation map of that filter. As a result, the network learns filters that activate when it detects some specific type of feature at some spatial position in the input. Stacking the activation maps for all filters along the depth dimension forms the full output volume of the convolution layer. CNNs share weights in convolutional layers, which means that all spatial locations share the same convolution kernel, which greatly reduces the number of parameters needed for a convolution layer. After each convolutional layer, it is conventional to apply a non-linear activation function immediately afterward. Deep CNNs with rectified linear units [*ReLUs*; *f*(*x*) = max(0, *x*)] train several times faster than their equivalents with tanh units (Krizhevsky et al., 2012).

#### Pooling Layer

It pools the activation of the neurons at one layer into a single neuron in the next layer. It can use two different pooling methods: max pooling and average pooling. Max pooling uses the maximum value from each cluster of neurons at the prior layer. Average pooling averages the value from each cluster of neurons at the prior layer. In the present work we used max pooling because it can boost signal from small regions of the image space and it is therefore best suited for our dataset, which includes very small lesions (in contrast, average pooling is more effective in the case of a large and noisy region of interest in the image). The pooling layer operates independently on every depth slice of the input and resizes it spatially. It serves two main purposes: (i) the number of parameters or weights is reduced, thereby decreasing the computational cost; and (ii) it controls over-fitting.

#### Fully Connected and Output Layers

In the fully connected layer every neuron is connected to all neurons in another layer. Finally, output layer neurons provide the prediction of the model.

#### CNN Implementation

**FIGURE 3:**
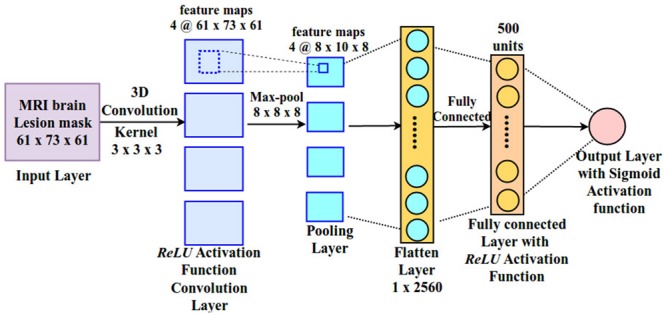
Architecture of the deep convolutional neural network (CNN).

The architecture of the CNN used in the present study is depicted in Figure 3. It includes one convolutional, one pooling, one fully connected and one output layer. The input layer is fed with a 3D lesion image (size: 61 × 73 × 61), followed by a 3D convolutional layer with four kernels (size: 3 × 3 × 3). *ReLU* activation function is applied on the convolutional layer and the output of this layer is passed to the pooling layer. 3D max pooling (8 × 8 × 8) is applied on the output of the convolutional layer, generating feature maps of size (8 × 10 × 8). The large pooling stride is motivated by the fact the size of the lesion is typically small and most of the image space is therefore occupied by zero values. Finally, activations are passed to the fully connected layer, consisting of 500 neurons with *ReLU* activation function, and then to the output layer. The output layer is made by a single neuron with sigmoid activation function, which represents the language score of the corresponding patient. This allows us to map the entire lesion image into a single (predicted) behavioral score.

Formally, the CNN implementation employed here can be described as follows. Consider a 3D MRI image x ∈ℜ^H ×W × D^ with *H* × *W* × *D* elements (height, width, and depth of an image), each of them indexed by a triplet (*i, j d*) with 0 ≤*i* < *H*, 0 ≤*j* < *W*, and 0 ≤*d* < *D*. *D* represents the number of slices in the MRI image. Each slice has *H* × *W* elements. Suppose we are considering the *l*th layer of a CNN, whose inputs form an order three tensor *x^l^* with x^l^ ∈ℜ^H_l_ × W_l_ × D_l_^. Thus, the triplet index set (*i^l^, j^l^, d^l^*) refers to one element in *x^l^* which is in the *d^l^*th slice at spatial location (*i^l^, j^l^*) (at the *i^l^*th row and *j^l^*th column). In the convolutional layer multiple kernels are used. Assuming *D* kernels are used and each kernel is of spatial span *H* × *W*, we denote all the kernels as *f*. *f* is an order four tensor ℜ*^H^*^×^*^W^*^×^*^D_l_^*^×^*^D^*. Similarly, we use indexed variables 0 ≤*i* < *H*, 0 ≤*j* < *W*, 0 ≤*d^l^* < *D^l^*, and 0 ≤*d* < *D* to pinpoint a specific element in the kernel. The basic flow of the CNN structure is represented by the following equation:

$$\begin{array}{c} x^{1}\to w^{1}\to x^{2}\ldots \to x^{L-1}\to w^{L-1}\to x^{L}\to w^{L}\to z \end{array}$$

The above equation illustrates how a CNN runs layer by layer in a forward pass. The input *x*^1^ goes through the processing in the first layer. We denote the weights involved in the first layer’s processing collectively as a tensor *w*^1^. The output of the first layer is *x*^2^, which also acts as an input to the next processing layer. This processing proceeds until output *x^L^*.

All CNN models used in this work were implemented in Tensorflow and were trained on GPUs using the Adam optimizer (Kingma and Ba, 2014; Abadi et al., 2015). Mean square error was used as loss function for training. All models were trained and tested using a LOOCV loop, which was also used to tune the hyperparameters.

### Hybrid Model (RR With CNN and PCA Features: f + RR)

We also assessed whether the features learned by the CNN at the top hidden layer provide information that is not captured by the PCA preprocessing used in the RR model. To this end, we trained a RR model where the features (neuron activations) encoded in the fully connected hidden layer of the CNN were added to the PCA-based features as input to the model.

### Quantifying Redundancy

Machine learning algorithms capture structure in the data that needs to be generalized in order to make predictions from new data. Therefore, the presence of similar data instances in the dataset is required for such models to work. However, these similarities must be defined in the most general manner to be effective on unseen examples. Trained models are obviously biased toward the kind of examples they have seen during training and images that have more similar examples in the data (redundancy) yield more accurate predictions. We explored how the redundancy of images, defined in terms of similarity (or distance metrics) would affect the predictive accuracy of the models. We defined three types of image-image distances for all pairs as follows:

#### Lesion Centroid Distance

Distance between two images was simply computed in terms of the Euclidean distance between the centroids of the lesions.

#### Topological Distance

This index is complementary to lesion-centroid and goes beyond the centroid comparison. The images are first centered at their lesion centroids and then images are cropped as per the larger lesion size, then every pair of voxels is compared in the transformed pair of images using Euclidean distance. The differences in voxel-wise comparisons is a measure of how the lesions differ in terms of topology irrespective of their centroid positions.

#### Location + Topological Distance

Since all MRI lesion images are aligned in a common reference frame, their unbiased topological differences can be measured by voxel to voxel signal differences. Direct comparison between images will also implicitly capture the location information as similar topologies at different locations will be computed as distant. We therefore computed the Euclidean distance between each pairs of images.

For each measure of distance between a pair of images a redundancy score (for each of the above definitions) was assigned to individual images. Since the models have been trained in a leave one out manner, the number of times an image occurs in similar pairs of images at a given threshold informs us about how much of training data is redundant with this image. A threshold was chosen as follows: in the full distance matrix, the number of column values lower than the (mean-SD) in the row is treated as the redundancy for the image label in that row. Row-wise thresholding was found to be more suitable as it not only represents the redundancy of the image but also it implicitly computes the range of distances of an image with all the others. After computing the redundancy of every image with reference to the rest of the data set, two groups of images with high and low redundancy were created and model performance was compared between them.

### Performance Metrics

Performance of the models was measured using the square of the Pearson correlation coefficient between actual and predicted scores (Siegel et al., 2016). Mean absolute error (MAE; i.e., the absolute difference between predicted and actual score) is also reported in some of the analyses.

## Results

The results presented below are divided into four sub-sections. We first look at the predictive accuracy of the different models/approaches. We then investigate how performance is affected by sample size and redundancy in the training dataset. Finally, we assess the model on prediction of chronic cognitive deficit as measured 3 months after the stroke.

### Overall Performance of Predictive Models on the Full Dataset

**FIGURE 4:**
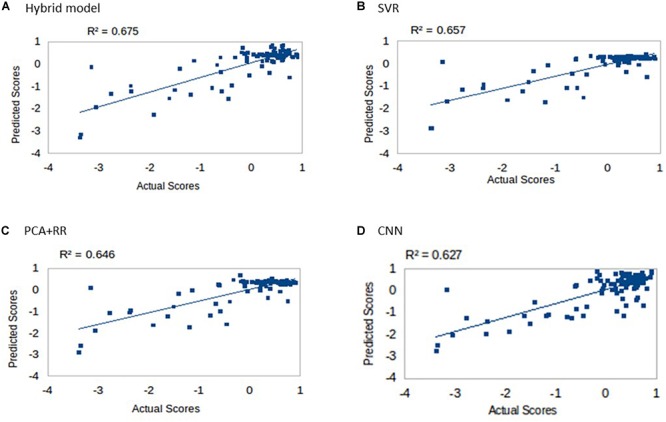
Language scores predicted by the four competing models: **(A)** Hybrid model, **(B)** SVR, **(C)** PCA + RR, and **(D)** CNN.

Multiple models were trained and tested on the dataset as described in the section “Materials and Methods” using LOO cross-validation (as in Siegel et al., 2016). Results for the four different approaches are shown in Figure 4. All models explained more than 60% of the variance and rank them according to the *r*^2^ values (in parentheses) produced the following order: Hybrid Model (0.675), SVR (0.657), PCA + RR (0.646), and CNN (0.627). Notably, the SVR model performed significantly better than CNN (*p* = 0.0402, two-tailed) and PCA + RR (*p* = 0.0001, two-tailed) in the comparison of *r*^2^ values. Therefore, it appears that deep learning did not lead to performance gains when evaluated against the two conventional, shallow learning methods. However, the Hybrid RR model, trained with composite features from PCA and CNN (the latter corresponding to neuron activations in the fully connected hidden layer) outperformed all other methods (*p* = 0.0001, two-tailed, for SVR vs. f + RR). These observations suggest that for the current dataset, which includes a relatively small number of samples, avoiding over-fitting is a key factor. A linear method like RR is therefore the most powerful when exploiting the rich set of features derived from both PCA and CNN learning on the lesion images. We will return to the issue of dataset size in the next section.

**FIGURE 5:**
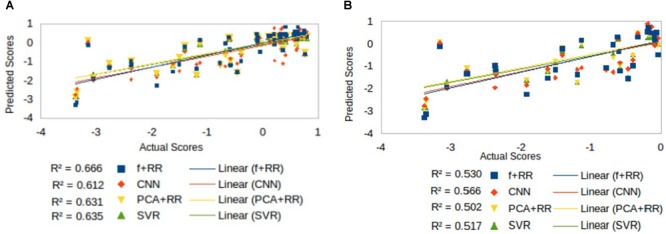
Comparison of models’ predictive accuracy on selected subsets of patients: **(A)** patients with left hemisphere lesions (*N* = 57), that is the population in which a language deficit is most common after stroke, and **(B)** patients with language deficit (*N* = 29) as attested by a score < 0.

Inspection of Figure 4 suggests that accuracy in predicting a specific range of scores does not necessarily reflect the ranking of the models’ overall predictive accuracy. For example, the CNN appears to resolve quantitative differences among language deficits patients despite the overall poorer model fit. We performed two supplementary analyses to further investigate the predictive accuracy of the models from this perspective. First, since language deficits are very uncommon following right hemisphere stroke, we compared the different models on the subset of patients with left hemisphere lesions (*N* = 57). Note that focusing on the population of left hemisphere stroke patients in relation to language deficits is a standard approach and it is well aligned with potential clinical applications of a computer model (Hope et al., 2013). The predictive accuracy of the models (see Figure 5A) was similar to that previously reported for the full dataset, thereby showing that the latter performance was not inflated by the inclusion of right hemisphere stroke patients. In the second analysis (see Figure 5B) we evaluated the models’ predictive accuracy across the range of scores that marks the presence of cognitive deficit, that is on the subgroup of patients (*N* = 29) who showed language deficit (score < 0). We found that the scores in the deficit range are better predicted by CNN than PCA + RR (*p* = 0.0425, two-tailed). These results suggest that the CNN model is better tuned to the fine-grained, quantitative prediction of the severity of deficit and help in explaining why CNN features boost the overall performance of the Hybrid model. For the sake of completeness, we also evaluated the models’ predictions on the subgroup of patients showing no language deficit (scores ≥ 0). Performance was very poor across models (all *r*^2^ values < 0.05). This is to be expected because individual differences within the range of unimpaired performance are independent of the nature of the lesions. Individual variability is expected also in the absence of lesions and it is obvious that it cannot be mapped onto a lesion image in the current framework. In summary, CNNs appear to extract useful high-level features that capture the association between 3D lesion images and language deficit scores.

### Role of Dataset Size on Predictive Accuracy

**FIGURE 6:**
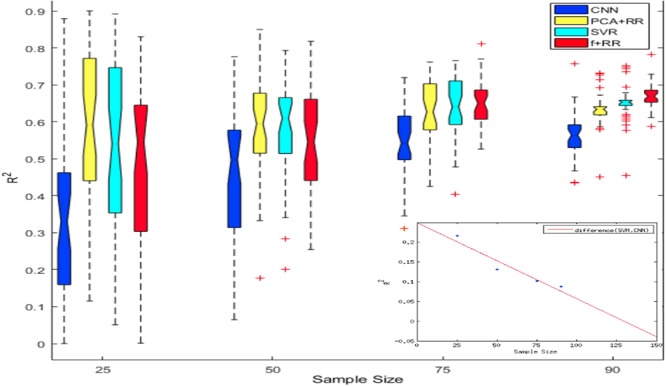
Notched box plot showing the prediction performance (*R*^2^) on 40 runs for each sample size and method. At the smaller sample sizes the performance levels of CNN or Hybrid models are either poorer or statistically similar to that of SVR and PCA-RR models (overlapping notches of the boxplot). However, at sample size of 90 the Hybrid model outperformed all other models. The inset plots the CNN performance gap (difference in *R*^2^ values) with increasing sample size in comparison to the SVR model; the best fitting function (red line) is extrapolated up to a sample size of 150.

Despite the favorable performance of CNNs in predicting the severity of deficit, the limited size of the dataset is likely to represent a crucial bottleneck. The issue of dataset size in MRI image analysis and prediction has been highlighted both in relation to the lesion-behavior mapping problem (Price et al., 2017) as well as for other types of medical imaging problems (Cho et al., 2015). Deep learning methods are highly effective when the number of samples available for training is large (Russakovsky et al., 2015; Shen et al., 2017). To investigate this issue in a more systematic way, we assessed how performance changed as a function of dataset size. We created multiple cohorts of four differently sized groups of patients (specifically 25, 50, 75, and 90 patients groups were created), which were randomly sampled from the full dataset. Random sampling was performed 40 times for each sample size. The models were then independently trained on all cohorts of the four datasets to assess generalization performance (LOO cross validation). Results of these simulations are presented in Figure 6. As can be noted, CNN’s performance is overall poorer than PCA + RR and SVR. This gap is especially clear for the smaller sample sizes, but it remains statistically significant even for the largest one (*p* = 0.036, two-tailed, for SVR vs. CNN at size 90). Nevertheless, the different models show markedly different patterns in terms of the effect of sample size. While PCA + RR and SVR models are relatively unaffected by sample size, performance of the CNN (as well as of the related Hybrid model) show large improvements with increasing sample size (see inset in Figure 6 for a plot of the performance gap of CNN with respect to SVR). These results suggest that the dataset size requirement for optimal performance of deep learning methods has not yet been satisfied and CNNs might significantly outperform competing models when more data will become available. By extrapolating the differential performance plot, we speculate that a few hundred samples may be needed for the CNN to outperform the SVR model. Note, however, that this comparison is relevant only for the conventional CNN approach, because we also established that the use of (hidden) features extracted by a CNN within a Hybrid (RR-based) model leads to be best predictive performance, presumably avoiding over-fitting caused by training them within the standard CNN framework (as shown in Figure 3).

### Role of Data Redundancy on Predictive Accuracy

**FIGURE 7:**
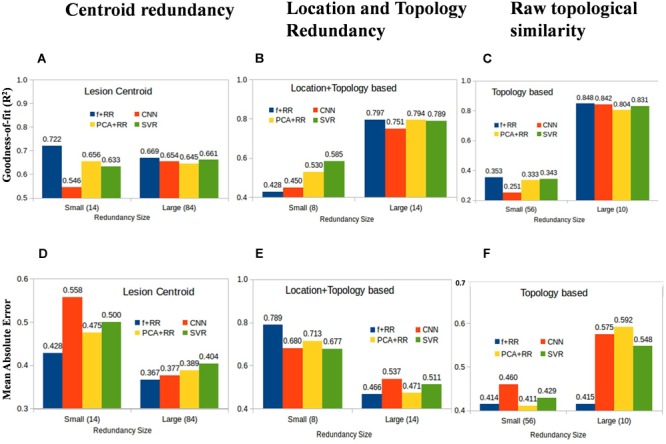
Average prediction performance for images having large vs. small redundancy levels with other images. Performance is measured by both goodness of fit (*r*^2^) **(A–C)** and mean absolute error (MAE) **(D–F)**. For these definitions of redundancy, almost all models perform very similarly in the image set in which redundancies are high (right-sided bars in all panels). In the small redundancy set, the Hybrid model comprehensively outperformed all other models, except in the location + topology based similarity. Values in the brackets of *x*-axis labels represent the number of images included in small and large redundancy groups.

Machine learning models acquire knowledge through exposure to the training data and are therefore sensitive to biases that may arise from examples that are over- or under-represented in the dataset. This also applies to a regression problem like the present one because some lesion patterns are likely to be more frequent and might therefore lead to better prediction than less frequent (or even unique) lesion patterns. However, the effect of the similarities between lesions might differ across models, particularly because the number of training parameters is different in each model. We defined three types of image redundancy indexes based on (a) lesion centroid distances (*centroid redundancy*); (b) lesion pixel-wise topological distances (*location + topology redundancy*); and (c) distances between directly superimposed images (*raw redundancy*) as described in the section “Materials and Methods.” Then, we grouped the images into two sets with high vs. low redundancy levels and we assessed the predictive accuracy of all models on these two image datasets. Figure 7 shows the performance of the smaller and larger clusters defined by redundancy levels in each category. This analysis provides insights into the lesion-predicted language deficits as follows.

#### Redundant Models Perform Better

All models show marked differences in predictive accuracy on the high vs. low redundancy image sets. The models perform very similarly in the image set in which redundancies are high. Not surprisingly, a model which has previously seen similar examples during training is much more accurate in prediction. This is consistent with the large data set requirements that were found to be critical for developing robust models as discussed in the previous section.

#### Hybrid Models Perform Better in Most Comparisons

In the small redundancy set, the Hybrid model comprehensively outperformed all other models, except when redundancy was defined using location + topology similarity. The finding that the Hybrid model performs better on images with limited redundancy is consistent with the idea that the lesion features extracted by the CNN are useful for prediction and complementary to those extracted by the PCA method.

#### Topology and Location Based Low Redundancy Produce Somewhat Competing Models

In the case of redundancy being defined by the location + topology similarity between images, performance on the low redundancy set was lower for the Hybrid model compared to the other models (Figure 7B,E). This suggests that the PCA and CNN features model contrasting properties in this comparison when redundancy is completely eliminated, and strictly non-redundant samples are left for training. We suggest that suboptimal models trained by CNN and PCA are independent of each other due to multiple and distinct sub-optimal solutions with similar performance in a high dimensional space. One explanation for this result is that highly generalizable features are needed for models to work well on data with low redundancy level. CNN and PCA-RR models generalize in different ways because data sets are small and multiple solutions with a similar (low) performance may emerge from learning on a large feature set. If that is the case, PCA and CNN-derived features may be inconsistent with each other, which in turn is detrimental to overall predictive performance. Note that this does not apply to the high redundancy set because there is a much smaller space of possible (good) solutions and features driving the prediction are likely to be more similar across learning methods. When redundancy is defined only along a single dimension, either location (centroid similarity) or topology (raw similarity), the low redundancy set still retains images that are redundant on the other dimension. The CNN-derived and PCA features will be therefore more similar and combining them in the Hybrid model improves performance. In summary, we find that redundancy defined in simple terms as variants of Euclidean distances of lesions is a critical parameter that determines how accurately a given model can predict language deficit based on MRI lesion images. Even though these results are obtained for the current data set, they are likely to be general in nature and it would be interesting to examine other MRI diagnostic problems in this context.

### Can Long Term Language Deficits Also Be Predicted?

**FIGURE 8:**
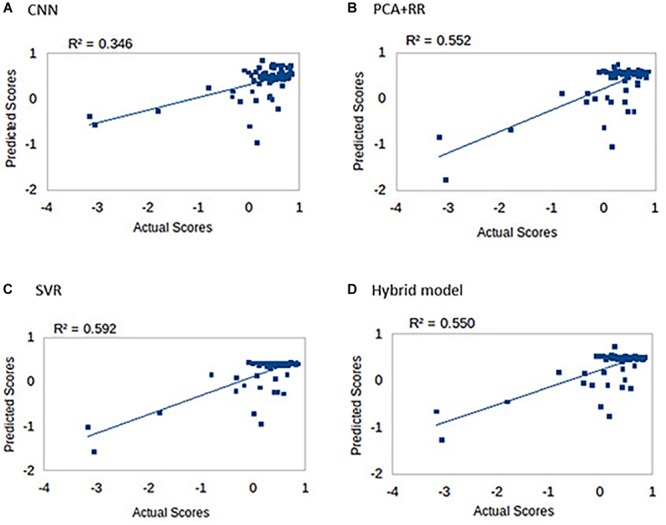
Long-term language scores predicted by the four machine learning models: **(A)** CNN, **(B)** PCA + RR, **(C)** SVR, and **(D)** Hybrid model. Predictive accuracy is indexed by R^2^ values.

One of the most interesting clinical applications of a computer model connecting brain lesion images to behavioral outcomes is the prediction of long term deficits. We therefore assessed all models in terms of the ability to predict the language score obtained in follow-up re-testing performed 3 months after the stroke (Ramsey et al., 2017). Indeed, Ramsey et al. (2017) reported that lesion topography (represented by PCA components of lesion images) accounted for about 13% of unique variance in the prediction of language recovery using regression analyses. Figure 8 summarizes our results (methods identical to the main analysis) on all patients (*N* = 74) for whom the long-term deficit scores were available.

Prediction accuracy for long-term deficit in terms of *r*^2^ values (range 0.35–0.59) was lower compared to our previous results (range 0.63–0.68). However, accuracy is remarkable when considering that the models did not include patients’ demographic data and/or acute-phase neuropsychological scores as (additional) predictors (see Hope et al., 2013; Ramsey et al., 2017).

## Discussion

In this work, we assessed deep and shallow machine learning approaches to predicting cognitive deficits from MRI lesion images. Conventional (shallow) machine learning methods typically require extraction and selection of image features that represent topological information about the lesion, a critical step that is dispensed with in the deep learning approach. We compared SVR and CNN techniques to a previously developed method based on RR. We also developed a Hybrid method based on re-using CNN’s high-level features together with PCA image features as input to a RR model, which yielded the best performance.

Overall, our results suggest that deep learning leverages predictive performance, which also scales up favorably with the amount of training data. Dataset size has been highlighted as a key issue for the lesion-behavior mapping problem (Price et al., 2017). Though the size of our dataset was far from optimal for deep learning, our analyses suggest that CNNs are likely to significantly outperform competing models when more patient data will become available. Moreover, we observed that the CNN already outperforms conventional models in resolving quantitative differences among the subgroup of patients with language deficit. This is crucial in the context of predicting the *severity of deficit* (i.e., a regression problem) as opposed to the mere *presence of deficit* (i.e., a classification problem). The CNN’s tuning to fine-grained prediction of the severity of deficit also helps explaining why CNN features boosted the overall performance of the Hybrid model.

We also systematically examined how predictive accuracy is influenced by data redundancy, defined in terms of similarity across lesion images using several distance metrics. Our analyses revealed that training on a dataset that contains multiple instances of similar lesions is a crucial factor to obtain good performance: lesion patterns that are more frequent lead to better prediction than less frequent lesion patterns. This is in line with the view that limited sample size is the main bottleneck for neuroimaging-based prediction of brain disorders (Arbabshirani et al., 2017).

It is worth noting that the performance gains obtained with deep learning come at the expense of interpretability. Conventional machine learning models can be readily analyzed to assess which image features (i.e., which voxels) are particularly weighted in computing the prediction (see Siegel et al., 2016). Whether similar results can be obtained from methods that analyze deep networks in terms of function of intermediate feature layers (e.g., Zeiler and Fergus, 2014) or hidden neurons’ receptive fields (e.g., Testolin et al., 2017) is an issue for future work. Conversely, deep learning might also be exploited to use raw MRI images (rather than lesion images) as input for predicting behavioral deficits; however, stroke lesion segmentation remains a challenging problem^1^ and manual delineation remains the gold standard. Given the limits of dataset size, design of an end-to-end pipeline might benefit from a transfer learning approach (see Wang S. et al., 2019; Wang S.H. et al., 2019 for applications to neuroimaging).

Other avenues for future research include the assessment of deep learning models that include connectivity data to address the question of whether predictive accuracy is leveraged by information on structural and/or functional disconnection among brain regions (Forkel et al., 2014; Siegel et al., 2016; Hope et al., 2018). Finally, the prospect of predicting long-term deficits and/or the potential for functional recovery has profound implications for clinical practice.

## Ethics Statement

The dataset used in the present work was obtained from a study on stroke patients carried out at the Washington University School of Medicine. The study and all procedures were approved by the Washington University School of Medicine Internal Review Board; written informed consent was obtained from all participants in accordance with the Declaration of Helsinki.

## Author Contributions

MZ, SA, and SC designed the study. SA, SC, and MZ wrote the manuscript. MC contributed to the interpretation of the datasets and results. SC performed the numerical simulations and analyses with the help and guidance of LV and SA. MD performed the dataset preparation. All authors discussed the results and reviewed final version of the manuscript.

## Conflict of Interest Statement

LV was employed by company Tata Consultancy Services (TCS Research), India. The remaining authors declare that the research was conducted in the absence of any commercial or financial relationships that could be construed as a potential conflict of interest.

## Footnotes

1 http://www.isles-challenge.org/
